# Prevalence and Diagnosis of Latent Tuberculosis Infection in Young Children in the Absence of a Gold Standard

**DOI:** 10.1371/journal.pone.0164181

**Published:** 2016-10-26

**Authors:** Tomas Maria Perez-Porcuna, Hélio Doyle Pereira-da-Silva, Carlos Ascaso, Adriana Malheiro, Samira Bührer, Flor Martinez-Espinosa, Rosa Abellana

**Affiliations:** 1 Departament de Salut Pública, Facultat de Medicina, Universitat de Barcelona, Barcelona, Catalunya, Spain; 2 Servei de Pediatria, CAP Valldoreix, Unitat de Investigació Fundació Mútua Terrassa, Hospital Universitari Mútua Terrassa, Terrassa, Catalunya, Spain; 3 Institut d'Investigacions Biomèdiques August Pi i Sunyer, Barcelona, Catalunya, Spain; 4 Laboratório Multidisciplinar - Fundação de Hematologia e Hemoterapia do Amazonas, Manaus, Amazonas, Brazil; 5 Instituto de Ciências Biológicas, Universidade Federal do Amazonas, Manaus, Amazonas, Brazil; 6 Instituto de Patologia Tropical e Saúde Pública, Universidade Federal de Goiás, Goiás, Goiânia, Brazil; 7 Fundação de Medicina Tropical Dr. Heitor Vieira Dourado (FMT-HVD), Manaus, Amazonas, Brazil; 8 Instituto Leônidas e Maria Deane - Fiocruz Amazônia, Fundação Oswaldo Cruz, Manaus, Amazonas, Brazil; Agencia de Salut Publica de Barcelona, SPAIN

## Abstract

**Introduction:**

For adequate disease control the World Health Organization has proposed the diagnosis and treatment of latent tuberculous infection (LTBI) in groups of risk of developing the disease such as children. There is no gold standard (GS) test for the diagnosis of LTBI. The objective of this study was to estimate the prevalence of LTBI in young children in contact with a household case of tuberculosis (TB-HCC) and determine the accuracy and precision of the Tuberculin Skin Test (TST) and QuantiFERON-TB Gold in-tube (QFT) used in the absence of a GS.

**Methods:**

We conducted a cross-sectional study in children up to 6 years of age in Manaus/Brazil during the years 2009–2010. All the children had been vaccinated with the BCG and were classified into two groups according to the presence of a TB-HCC or no known contact with tuberculosis (TB). The variables studied were: the TST and QFT results and the intensity and length of exposure to the index tuberculosis case. We used the latent class model to determine the prevalence of LTBI and the accuracy of the tests.

**Results:**

Fifty percent of the children with TB-HCC had LTBI, with the prevalence depending on the intensity and length of exposure to the index case. The sensitivity and specificity of TST were 73% [95% confidence interval (CI): 53–91] and 97% (95%CI: 89–100), respectively, versus 53% (95%CI: 41–66) and 81% (95%CI:71–90) for QFT. The positive predictive value of TST in children with TB-HCC was 91% (95%CI: 61–99), being 74% for QFT (95%CI: 47–95).

**Conclusions:**

This is one of the first studies to estimate the prevalence of LTBI in children and the parameters of the main diagnostic tests using a latent class model. Our results suggest that children in contact with an index case have a high risk of infection. The accuracy and the predictive value of the two tests did not significantly differ. Combined use of the two tests showed scarce improvement in the diagnosis of LTBI.

## Introduction

Tuberculosis (TB) is one of the most prevalent contagious infections worldwide[1]. Nonetheless, although the incidence of TB is decreasing by approximately 1.5% annually, according to the elimination target set for 2050 proposed by the World Health Organization (WHO) this reduction is insufficient[2]. This may be explained by the underdiagnosis of the disease which has been estimated to be around one third of the incidence cases[1]. For adequate disease control the WHO has proposed the need for not only adequate diagnosis and treatment of TB cases but also the diagnosis and treatment of latent tuberculous infection (LTBI) in groups of risk of developing the disease such as children[2–4]. Most cases of pediatric age are due to household contact with adults with TB[5,6]. Therefore, pediatric control measures involve contact screening to detect and treat children with TB and LTBI in order to prevent the disease in those infected[7].

The tuberculin skin test (TST) and Interferon-γ release assays (IGRAs) are used for the diagnosis of LTBI and are based on challenging the immune system in order to detect whether it recognizes the antigens of *Mycobacterium tuberculosis* (Mtb)[8,9]. However, relatively few studies have been carried out in children, and the results described suggest that the performance of these assays differs from those obtained in adults, especially in countries with a high incidence of TB and TB in young children[10]. The absence of a reference test for these techniques makes their evaluation and comparison difficult[8]. With the latent class model defined by Dendukuri and Joseph (2001) the prevalence and the performance parameters of several diagnostic tests without gold standard can be estimated[11].

The objective of the present study was to estimate the prevalence of LTBI and determine the accuracy and safety of the TST and QuantiFERON-TB Gold In-Tube (QFT) tests in young children taking into account the intensity of exposure of the children to an index case. The population studied included children up to 6 years of age vaccinated with the BCG in Manaus (Brazil) during the years 2009–2010.

## Material and Methods

### Study design and population

We conducted a cross-sectional study of children to calculate the prevalence and accuracy of the TST and QFT. The study included a sample of children of up to 6 years of age without recent known contact with an index TB case and another group of children in contact with an index case within the previous 12 months. Case recruitment was performed in the *Policlínica Cardoso Fontes* (regional reference center for TB) and in the *Fundação de Medicina Tropical Dr*. *Heitor Vieira Dourado* (FMT-HVD), Manaus, Amazonas, Brazil from March 2009 to February 2010. Adults (greater than 12 years of age) diagnosed with TB in both centers were questioned about contact with children from 0 to 6 years of age. Those responding affirmatively were asked to bring the children to the center for evaluation and were invited to participate in the study. All the adult index cases were sputum smear and/or culture positive. Subjects receiving treatment or prophylaxis for TB were excluded. The controls were selected among children attended in both centers for reasons other than TB. The study was undertaken at an outpatient level.

### Ethics Statement

The study was approved by the Ethical Committee in Investigation of the FMT-HVD, October 26, 2007 (Protocol 2865–07). The legal guardians of all the participants provided written informed consent for inclusion in the study.

### Data collection

Demographic data as well as the epidemiologic history of exposure to an index case of TB and the clinical history of the patient and physical examination were recorded. Chest X-ray, the TST, and blood analysis (prior to the TST) were performed. The human immunodeficiency virus (HIV) test was not obligatory but was recommended to all the participants.

The *Mycobacterium tuberculosis* contact score (MTC-score) was used to evaluate the intensity of exposure. The MTC-score is from 0 to 15 and is based on the assumption that the gradient of Mtb exposure is a composite function of the infectivity of the index case (0–4), the duration of exposure in hours per day (0–4), the relationship to the index case (0–4) and the type of exposure (0–3)[12].

The time (months) from symptom onset to the time of initiation of treatment of the index case was calculated to measure the total exposure time (contagion) of the child to the index case.

### Procedures

The TST was performed with an intradermic injection of 2 tuberculin units (TU) of PPD RT23 (Statens Serum Institut, Copenhagen, Denmark) and read 72 hours thereafter. A strong TST reaction (TST+) was defined when induration was ≥10 mm [7].

The QFT test (Cellestis, Carnegie, Australia) was carried out and interpreted according to the manufacturer’s instructions by experienced laboratory technicians who were unaware of the data of the study subjects.

### Statistical analysis

Categorical variables were described using absolute frequencies and percentages and quantitative variables using the 25, 50 and 75 percentiles. The chi-square test was used to study the association between the results of the two tests and the variables of gender and age groups (less than or equal to 24 months and greater than 24) and the Mann Whitney U test was used for the exposure time and the MTC-score variables. Concordance between the QFT and the TST tests was measured by the kappa index.

The statistical analysis was divided into two phases. In the first phase the accuracy of the two tests was estimated, and the prevalence of LTBI was determined based on whether the children had been in contact with an index case or not. To estimate these parameters the proposal of Dendukuri N. and Joseph L. (2001) was used[11], stratifying the model to estimate the prevalence of LTBI in children with and without contact with an index case. Then, it was assumed that the combinations of both test results were distributed by a multinomial distribution with probabilities equal to:

$$\text{P}\left(\text{QFT}=+,\text{ TST}=+\right)=P_{i}\;\cdot \;\left[\left({\text{S}}_{1}\cdot {\text{S}}_{2}\right)+{\text{ covS}}_{12}\right]+\;\left(1-P_{i}\right)\cdot \;\left[\left(\left(1\;-{\text{ C}}_{1}\right)\cdot \left(1\;-{\text{ C}}_{2}\right)\right)+{\text{ covC}}_{12}\right]$$

$$\text{P}\left(\text{QFT}=+,\text{ TST}=-\right)=P_{i}\;\cdot \;\left[\left({\text{S}}_{1}\;\cdot \left(1\;-{\text{ S}}_{2}\right)\right)-{\text{ covS}}_{12}\right]\;+\;\left(1-P_{i}\right)\;\cdot \;\left[\left(\left(1\;-{\text{ C}}_{1}\right)\;\cdot \;{\text{C}}_{2}\right)-{\text{ covC}}_{12}\right]$$

$$\text{P}\left(\text{QFT}=-,\text{ TST}=+\right)=P_{i}\;\cdot \;\left[\left(\left(1\;-{\text{ S}}_{1}\right)\;\cdot {\text{ S}}_{2}\right)\;-{\text{ covS}}_{12}\right]+\;\left(1-\;P_{i}\right)\;\cdot \;\left[\left({\text{C}}_{1}\;\cdot \;\left(1\;-{\text{ C}}_{2}\right)\right)-{\text{ covC}}_{12}\right]$$

$$\text{P}\left(\text{QFT}=-,\text{ TST}=-\right)=P_{i}\;\cdot \;\left[\left(\left(1\;-{\text{ S}}_{1}\right)\;\cdot \;\left(1\;-{\text{ S}}_{2}\right)\right)\;+{\text{ covS}}_{12}\right]\;+\;\left(1\;-\;P_{i}\right)\;\cdot \;\left[\left({\text{C}}_{1}\;\cdot \;{\text{C}}_{2}\right)+{\text{ covC}}_{12}\right]$$

Where P_i_ is the prevalence of LTBI for the i*th* stratum (1: children with contact with an index case, and 2: without contact with an index case), S_1_ and S_2_ are the sensitivity of QFT and the TST test respectively, and C_1_ and C_2_ are the specificities. CovS_12_ and covC_12_ are the covariance between tests among subjects with latent infection and non-latent infection, respectively.

Both prevalences were assumed to have a Beta(1,1) prior distribution. Based on the literature of LTBI, we reasonably considered that the sensitivity and the specificity of the two tests lie within the range of 50%-100%. Consequently, the prior distribution for these parameters was a Beta(8.25, 2.75). The covariance parameters are taken to have Beta(1,1) prior distribution, and their feasible ranges were given as 0 ≤ *covS*_12_ ≤ min(*S*_1_,*S*_2_) − *S*_1_*S*_2_ and 0 ≤ *covC*_12_ ≤ min(*C*_1_,*C*_2_) − *C*_1_*C*_2_,

According to the proposal of Martinez E.Z. (2008),[13], in the second phase the previous model is widened and rather than study the prevalence based on whether there was or was not exposure to an index case it is studied based on the magnitude of intensity and the time of exposure to an index case. Denoting the true infection status (latent) of the k*th* subject as D_k_ (0: among non-infected subjects and 1 among infected subjects), the prevalence of LTBI is the probability of being truly infected P(D_k_ = 1), and it is given as covariates:

$$P\left(D_{k}=1\right)=\text{Φ}\left(\beta_{0}+\beta_{1}\times ExpositionTime+\beta_{2}\times MTC-Score\right)$$

Φ: represents the cumulative distribution function of the Normal(0,1) distribution

The conditional dependence between tests is modeled using a random effects model[14]. In this case, the sensitivities and specificities of the tests are modeled as a function of subject-specific random effect, i_k_. Thus, the probability that the k*th* subject has a positive result in the j*th* test (1: QFT and 2: TST) is given as:

$$P\left(T_{jk}=+|D_{k}=1,I_{k}=i_{k}\right)=\text{Φ}\left(\beta_{3j}+b_{1}i_{k}\right)$$

And has a negative result

$$P\left(T_{jk}=-|D_{k}=0,I_{k}=i_{k}\right)=\text{Φ}\left(\beta_{4j}+b_{0}i_{k}\right)$$

where:

i_k_: is the intensity of the *k*th subject, and it is a random variable following a Normal(0,1) distribution.

Thus, the mean sensitivity of the *jth* test in all the subjects was:

$$S_{j}=P\left(T_{j}=+|D=1\right)=\overset{\infty }{\underset{-\infty }{\int }}P\left(T_{jk}=+|D_{k}=1,I_{k}=i_{k}\right)d\text{Φ}\left(i_{k}\right)=\text{Φ}\left(\frac{\beta_{3j}}{\sqrt{1+b_{1}^{2}}}\right)$$

and the mean specificity of the *jth* test in all the subjects was:

$$C_{j}=P\left(T_{j}=-|D=0\right)=\overset{\infty }{\underset{-\infty }{\int }}P\left(T_{jk}=-|D_{k}=0,I_{k}=i_{k}\right)d\text{Φ}\left(i_{k}\right)=\text{Φ}\left(-\frac{\beta_{4j}}{\sqrt{1+b_{0}^{2}}}\right)$$

Using the estimates obtained in the first phase of the prevalence, sensitivity, specificity and the covariance between test, the parameters of the prior distributions from the constants of the model were β_0_~Normal(-1.59,1.8), β_31_~Normal(0.24,0.32), β_32_~Normal(0.83,0.49), β_41_~Normal(1.15,0.32) and β_42_~Normal (2.01,0.48) and for the b_1_ and b_0_ were Normal(0.77,0.5) and Normal(0.99,0.5), respectively. The prior distribution for the coefficient of the exposure time and MTC-score was a Normal (0,0.1), respectively[15].

## Results

**Fig 1:**
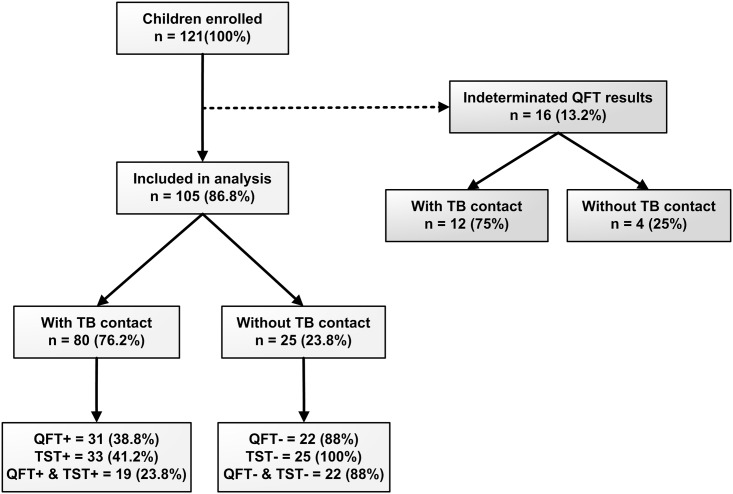
Flow diagram of enrollment. TB: tuberculosis, QFT: QuantiFERON-TB Gold In-Tube, TST: Tuberculin Skin Test.

A total of 121 children were studied, 92 (76.0%) of whom had had contact with an index case and 29 (24.0%) had not. All the children studied had received the BCG vaccination in the first 2 months of life. Sixteen children (13.2%) were excluded from the analysis because they presented an indeterminate result with QFT. Of the 105 children analyzed, 41.9% were male and 23.8% were less than 24 months of age. Twenty-five children (23.8%) had had no contact with an index case but in 3 children result was positive for the QFT test but not for the TST test (Fig 1). There were no significant differences when comparing age and gender in exposed children and controls. Of the 80 (76.2%) children having contact with an index case, the median MTC-score was 12.0 points (IQR = 3.75), and the time of exposure was 2 months (IQR = 4.0). Thirty-one children (38.8%) showed a positive QFT result and 33 (41.2%) did so with the TST, but only 19 children (23.9%) were positive for both tests. None of the positive TST results were weak (between 5–9 mm). The two tests showed a low concordance of 0.364 (p< 0.001).

The results of the two tests were not associated with either the gender or age of the children. The time and the intensity of the exposure were associated with the QFT and TST results (Table 1). The time and the intensity of the exposure of the children with a positive QFT result (median time of exposure = 2; median MTC-score = 12.0) was greater than that of children with a negative QFT (median time of exposure = 1.0; p = 0.026; median MTC-score = 10.0, p = 0.021). The time and the intensity of the exposure of the children with a positive TST result (median time of exposure = 4; median MTC-score = 12.0) was greater than in those with a negative TST result (median time of exposure = 1.0, p<0.001, median MTC-score = 9.5, p<0.001).

**Table 1:** Descriptive analysis and comparisons between the results of the QFT and the TST test in children up to 6 years of age in Manaus, Brazil.

	QFT			TST		
	+	-	p-value	+	-	p-value
Male	14 (31.8%)	30 (68.2%)	0.917	14 (31.8%)	30 (68.2%)	0.942
Female	20 (32.8%)	41 (67.2%)		19 (31.1%)	42 (68.9%)	
Age< = 24 months	7 (28.0%)	18 (72.0%)	0.592	8 (32.0%)	17 (68.0%)	0.944
Age >24 months	27 (33.8%)	53 (66.2%)		25 (31.2%)	55 (68.8%)	
Age (months)*	47.76 (22.5)	43.68 (21.5)	0.384	43.91 (22.7)	45.50 (22.4)	0.737
Exposure Time**	2 (5.25–1)	1 (0–3)	0.026	4 (1–10)	1 (0–3)	<0.001
MTC-score**	12 (14.0–8.75)	10 (0–12)	0.021	12 (10–14)	9.5 (0–12)	<0.001

* mean (standard deviation). Student t.

**median (quartile 25-quantile 75). Mann-Whitney U.

TST: Tuberculin Skin Test,QFT: QuantiFERON-TB Gold In-Tube, MTC-SCORE: *Mycobacterium tuberculosis* contact score.

### Bayesian Analysis

The prevalence of infection in the group of children without contact with an index case was 0.04 (95% CrI [0.00, 0.20], being 0.50 in children with contact (95% CrI [0.28, 0.81]). The sensitivity of the QFT test was 0.58 (95% CrI [0.41, 0.78]) and 0.75 (95% CrI [0.49, 0.94]) for the TST test. The specificity of the QFT was 0.79 (95% CrI [0.67, 0.91]) and 0.92 (95% CrI [0.78, 0.98]) for the TST test (Table 2). The positive predictive values of both tests were very low in the group of children with no contact with an index case and thus, the prevalence of infection was very low. In the group of children with contact with an index case, the probability of a child diagnosed as positive really being infected was 0.74 (95% CrI [0.47, 0.95]) for QFT and 0.91 (95% CrI [0.61, 0.99] for TST. Moreover, the probability of a child diagnosed as negative really not being infected was 0.65 (95% CrI [0.27, 0.88]) for QFT and 0.79 (95% CrI [0.31, 0.96]) for TST. On evaluating the positive predictive value with the results of both tests together we found that the probability of really being infected when the two tests were positive was 0.93 (95% CrI [0.69, 0.99]). When only the QFT was positive this value was 0.35 (95% CrI [0.04, 0.90]), being 0.90 (95% CrI [0.30, 0.99]) when only the TST was positive. In relation to the negative predictive values, the probability of not being infected when both tests were negative was 0.83 (95% CrI [0.37, 0.97]), and when only the QFT was negative this value was 0.1 (95% CrI [0.01, 0.7]), being 0.65 (95% CrI [0.10, 0.96]) (Table 3) when only the TST was negative.

**Table 2:** Results of the prevalence of LTBI according to exposure to an index TB case and the sensitivity and the specificity of QFT and TST in children up to 6 years of age in Manaus, Brazil (first phase).

	Median	95% CrI
P(LTBI) Unknown exposure of children	0.04	0.00–0.20
P(LTBI) Exposed Children	0.50	0.28–0.81
Sensitivity of QFT	0.58	0.41–0.78
Specificity of QFT	0.79	0.67–0.91
Sensitivity of TST	0.75	0.49–0.94
Specificity of TST	0.92	0.78–0.98

CrI: Credibility interval, TST: Tuberculin Skin Test, QFT: QuantiFERON-TB Gold In-Tube, MTC-SCORE: *Mycobacterium tuberculosis* contact score, LTBI: latent tuberculosis infection.

**Table 3:** Probability of LTBI in children up to 6 years of age according to the results of both tests in the latent class model (first phase).

	Unknown exposure of children		Exposed Children	
	Median	95% CrI	Median	95% CrI
P(LTBI|QFT+)	0.01	0.00–0.51	0.74	0.47–0.95
P(LTBI|TST+)	0.27	0.01–0.79	0.91	0.61–0.99
P(LTBI|QFT+ TST+)	0.31	0.01–0.83	0.93	0.69–0.99
P(LTBI|QFT+ TST-)	0.02	0.00–0.34	0.35	0.04–0.90
P(LTBI|QFT- TST+)	0.29	0.01–0.82	0.90	0.31–0.99

CrI: Credibility interval, TST: Tuberculin Skin Test, QFT: QuantiFERON-TB Gold In-Tube, MTC-SCORE: *Mycobacterium tuberculosis* contact score, LTBI: latent tuberculosis infection.

**Fig 2:**
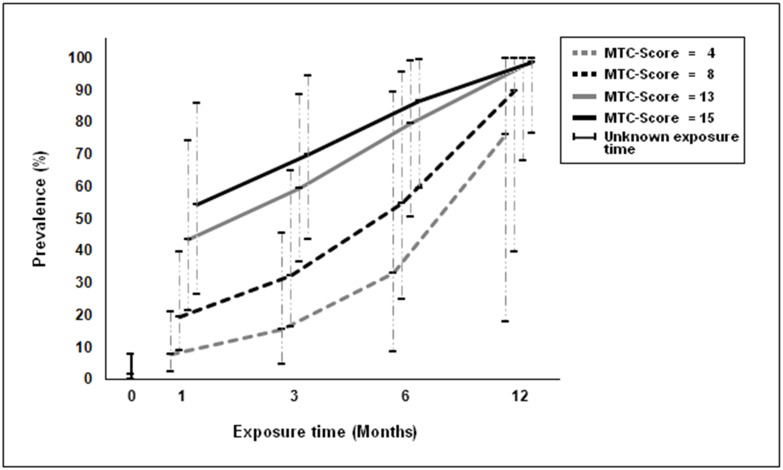
Prevalence of LTBI according to the time of exposure of 0, 1, 3, 6 and 12 months and the MTC-score values of 4, 8, 13 and 15 in children up to 6 years of age in Manaus, Brazil. MTC-SCORE: *Mycobacterium tuberculosis* contact score.

**Fig 3:**
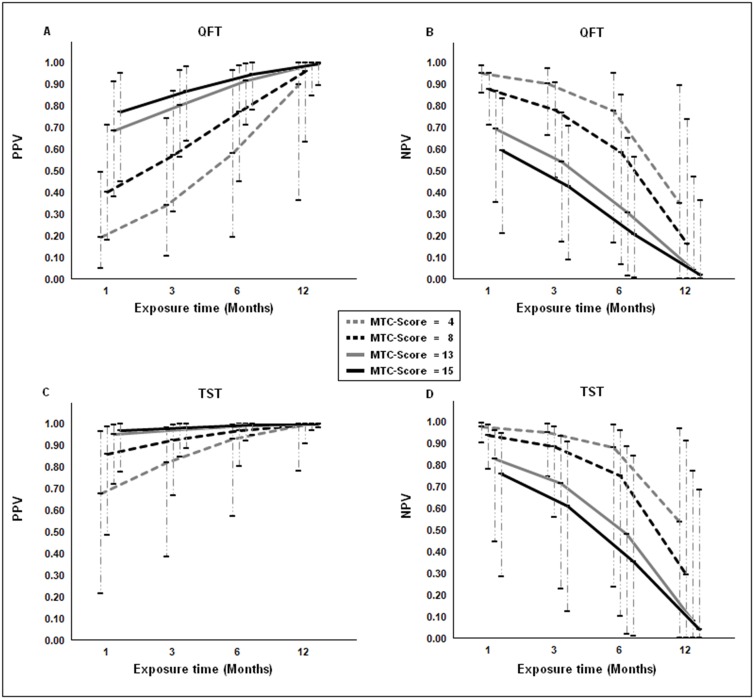
Positive and negative predictive values for QFT for times of exposure of 1, 3, 6, and 12 months and intensity of exposure of 4, 8, 13 and 15. TST: Tuberculin Skin Test, QFT: QuantiFERON-TB Gold In-Tube, PPV: positive predictive values, NPV: negative predictive values.

Taking into account the magnitude of the intensity (MTC-score) and the time of exposure, according to the model used, the prevalence of LTBI increased with the intensity (beta = 0.14, 95% CrI [0.06, 0.23]) and the time of exposure (beta = 0.19, 95% CrI [0.05, 0.49]) (Tables 4 and 5). Fig 2 shows the values of prevalence for the times of exposure of 0, 1, 3, 6 and 12 months and MTC-score values of 4, 8, 13 and 15. Children with an exposure time of 12 months and MTC-score greater than 8 presented prevalences greater than 75% while children with low exposure times (less than or equal to 1 month) and a MTC-score less than 5 presented prevalences of less than 25%. On taking into account the intensity and time of exposure in this model, the sensitivity and specificity of the tests was slightly modified. Fig 3A and 3B show the positive predictive values based on the intensity and time of exposure. The results indicate that with high exposure times (greater than or equal to 6 months) and exposures of greater than or equal to 13, the predictive value of QFT was greater than 0.70, being greater than 0.9 for TST. The negative predictive values of both tests showed a similar behavior, being high with a low time and intensity of exposure and low when the time and intensity were high (Fig 3C and 3D).

**Table 4:** Probability of non LTBI (LTBI^c^) in children up to 6 years of age according to the results of both tests in the latent class model (first phase).

	Unknown exposure of children		Exposed Children	
	Median	95% CrI	Median	95% CrI
P(LTBI^c^|QFT-)	0.98	0.88–1.00	0.65	0.27–0.88
P(LTBI^c^|TST-)	0.99	0.90–1.00	0.79	0.31–0.96
P(LTBI^C^|QFT+ TST-)	0.98	0.66–1.00	0.64	0.10–0.96
P(LTBI^c^|QFT- TST+)	0.76	0.18–0.99	0.10	0.01–0.69
P(LTBI^c^|QFT- TST-)	0.99	0.93–1.00	0.83	0.37–0.97

CrI: credibility interval, TST: Tuberculin Skin Test, QFT: QuantiFERON-TB Gold In-Tube, MTC-SCORE: *Mycobacterium tuberculosis* contact score, LTBI: latent tuberculosis infection.

**Table 5:** Estimation of the sensitivity and specificity of QFT and TST, and the coefficients of regression of the variables of time of exposure and MTC-Score associated with the prevalence (second phase).

Variable	Median	95% CrI
Exposure Time	0.19	0.05–0.49
MTC-Score	0.14	0.06–0.23
Sensitivity of QFT	0.53	0.41–0.66
Specificity of QFT	0.81	0.71–0.90
Sensitivity of TST	0.73	0.53–0.91
Specificity of TST	0.97	0.89–1.00

CrI: credibility interval, TST: Tuberculin Skin Test, QFT: QuantiFERON-TB Gold In-Tube, MTC-SCORE: *Mycobacterium tuberculosis* contact score, LTBI: latent tuberculosis infection.

## Discussion

Estimation of the prevalence of a disease and the parameters of sensitivity, specificity and predictive values of the tests used to study this disease in the absence of a gold standard are an important advance for the evaluation of diagnostic tests. This is of special relevance in the current epidemiological and diagnostic context of the study of LTBI in which it is impossible to determine if an individual is really infected or whether this person presents immune response to the previous infection.

The prevalence of LTBI in the group exposed to an index case was 50.4% (27.1%-83.3%), and this prevalence or probability of becoming infected increases with both the intensity (MTC-score) and the time of exposure to the index case[16]. The prevalence in the control group was 3.9% (0.10%-20.6%).

The prevalence obtained suggests that young children are very susceptible to exposure to an index case, with this prevalence possibly being greater than in other population groups[17]. On the other hand, most of the cases of LTBI and TB disease in young children are the result of exposure to a known close index case of TB[5]. These two reasons reinforce the crucial importance of diagnostic strategies of infection and disease in household pediatric contacts in order to control TB[18].

Knowledge of the prevalence of LTBI based on the time and intensity of exposure allows estimation of the prevalence and thus, the risk of LTBI in a young child before obtaining the result of the diagnostic tests. Indeed, we found that the high intensity and time of exposure result in very high prevalences (greater than 70%).

Since it is difficult to precisely know at what time an individual is contagious, we estimated the period of contagion from the time at which the index case shows symptomatology compatible with TB until the time of treatment. Likewise, to evaluate the intensity of exposure with the index case we used the MTC-score which assesses the capacity of contagion of the index case and the grade of relationship with the same. Our results suggest that the use of the time of exposure and MTC-score are feasible for study models of LTBI in children.

Concordance between the TST and QFT was lower than expected. This has been reported previously in young children, especially in regions with a high incidence of TB[19] and may be related to coinfection with helminths, malnutrition, short exposure times, immunosuppression and reversion phenomena in the IGRAs[16,20,21].

Despite the low concordance, the tests presented similar sensitivities and specificities, without significant differences. It is of note that the specificity of the TST was not lower than QFT. This result is surprising in view of the widespread opinion that the BCG vaccine produces false positives in the TST[22]. Some authors have suggested that this effect is lower in tropical regions[23], and the type of BCG strain vaccinated may also have different effects on the TST[24].

The predictive values which depend on the prevalence and accuracy of the tests did not show significant differences according to the test applied, despite the TST being superior in all the values. Likewise, the results obtained suggest that both tests have a very high predictive capacity when the intensity and time of exposure are high and are therefore useful for diagnosis in the study of contacts. In the case of controls we observed very low positive predictive values thereby indicating the scarce utility of these tests in population screenings [2].

On evaluating the results of the predictive values in the two tests combined it was found that these values in the two tests alone improved, albeit not significantly. According to the latent variable model, when the results between the tests were discordant, the TST presented a greater probability of correctly classifying the individual. Thus, in young children QFT results discordant with those of TST should be interpreted with caution since the predictive values fall markedly. This may explain, in part, the discordance observed between the two tests in short times of exposure similar to what has previously been described in this group[16].

The indeterminate results for QFT were high in our study. The proportion of indeterminate results varies greatly in studies in children [25–27] and may be associated with technical problems and probably also factors which alter Th1 immune response[28] such as age, the presence of helminths, and immunosuppression[29].

This study has several limitations. The relatively small sample size may have limited the power of the study to find significant differences. Nonetheless, there are few studies in small children and the results obtained are biologically logical. In addition, the sensitivity and specificity may vary widely among studies and this may affect the bayesian analysis. However, we have attempted to control this with the use of wide priors for the sensitivity and specificity of both tests. The third limitation was that we used the WHO definition of TST positivity (≥10 mm), and other cut off points might have shown different results.

On the other hand, the strengths of this study are: 1. There are few studies in small children who are the most vulnerable to *M*. *tuberculosis*. 2. The methodology used is novel in TB but has been well contrasted in other infectious diseases [30]. This study allows calculation of the prevalence of the disease which, to date, has not been done since LTBI cannot currently be microbiologically demonstrated due to its low bacillary load. Up to now both the prevalence and the accuracy of the tests has, in many cases, been calculated using an imperfect gold standard (one of the two tests) or has been extrapolated from ill children, who are clinically different from those who are infected. 3. The results obtained are biologically logical since the test results are correlated with the time and intensity of exposure.

## Conclusions

This is one of the first studies to estimate the prevalence of LTBI in children and the parameters of its main diagnostic tests using a latent class model. The study of LTBI in young children is of great importance due to the high possibility of developing the disease and increasing the reservoir of cases which these cases may represent in the future if not treated[5,6]. The results of the present study suggest that children in contact with an index case have a high risk of infection according to the intensity and length of exposure to the index case, and this should be taken into account in the design of TB control strategies.

The TST and QFT showed similar characteristics and behavior but concordance was lower than expected. Combined use of the two tests in our study showed scarce improvement in the diagnosis of LTBI. We know that diagnostic tests of LTBI are not perfect. However, the latent variable model allows better evaluation of these tests and estimation of the prevalence of LTBI, thereby facilitating the development of more adequate approaches to this crucial problem of TB control. The diagnosis of LTBI and disease is the key to the control of pediatric TB and requires more and greater efforts to this end.

## Abbreviations

BCG: Calmette-Guérin bacillus vaccine
CI: Confidence interval
IGRAs: Interferon-γ release assays
IQR: Interquartile range
HIV: Human immunodeficiency virus
LTBI: Latent tuberculous infection
Mtb: *Mycobacterium tuberculosis*
MTC-score: *Mycobacterium tuberculosis* contact score
PPD: Purified protein derivatives
QFT: QuantiFERON-TB Gold in-tube
TB: Tuberculosis
TB-HCC: Household case of tuberculosis
TST: Tuberculin Skin Test
TU: Tuberculin units
WHO: World Health Organization
